# *MaMYB4*, an R2R3-MYB Repressor Transcription Factor, Negatively Regulates the Biosynthesis of Anthocyanin in Banana

**DOI:** 10.3389/fpls.2020.600704

**Published:** 2021-01-07

**Authors:** Gui-Ming Deng, Sen Zhang, Qiao-Song Yang, Hui-Jun Gao, Ou Sheng, Fang-Cheng Bi, Chun-Yu Li, Tao Dong, Gan-Jun Yi, Wei-Di He, Chun-Hua Hu

**Affiliations:** ^1^Institute of Fruit Tree Research, Guangdong Academy of Agricultural Sciences/Key Laboratory of South Subtropical Fruit Biology and Genetic Resource Utilization (Ministry of Agriculture and Rural Affairs)/Guangdong Province Key Laboratory of Tropical and Subtropical Fruit Tree Research, Guangzhou, China; ^2^Key Laboratory of Horticultural Plant Biology of the Ministry of Education, College of Horticulture and Forestry Sciences, Huazhong Agricultural University, Wuhan, China

**Keywords:** banana, anthocyanin, R2R3-MYB repressor, MaMYB4, *bHLH*

## Abstract

Anthocyanins spatiotemporally accumulate in certain tissues of particular species in the banana plant, and MYB transcription factors (TFs) serve as their primary regulators. However, the precise regulatory mechanism in banana remains to be determined. Here, we report the identification and characterization of *MaMYB4*, an R2R3-MYB repressor TF, characterized by the presence of EAR (ethylene-responsive element binding factor–associated amphiphilic repression) and TLLLFR motifs. *MaMYB4* expression was induced by the accumulation of anthocyanins. Transgenic banana plants overexpressing *MaMYB4* displayed a significant reduction in anthocyanin compared to wild type. Consistent with the above results, metabolome results showed that there was a decrease in all three identified cyanidins and one delphinidin, the main anthocyanins that determine the color of banana leaves, whereas both transcriptome and reverse transcription–quantitative polymerase chain reaction analysis showed that many key anthocyanin synthesis structural genes and TF regulators were downregulated in *MaMYB4* overexpressors. Furthermore, dual-luciferase assays showed that *MaMYB4* was able to bind to the *CHS*, *ANS*, *DFR*, and *bHLH* promoters, leading to inhibition of their expression. Yeast two-hybrid analysis verified that MaMYB4 did not interact with bHLH, which ruled out the possibility that MaMYB4 could be incorporated into the MYB-bHLH-WD40 complex. Our results indicated that *MaMYB4* acts as a repressor of anthocyanin biosynthesis in banana, likely due to a two-level repression mechanism that consists of reduced expression of anthocyanin synthesis structural genes and the parallel downregulation of *bHLH* to interfere with the proper assembly of the MYB-bHLH-WD40 activation complex. To the best of our knowledge, this is the first MYB TF that regulates anthocyanin synthesis that was identified by genetic methods in bananas, which will be helpful for manipulating anthocyanin coloration in banana programs in the future.

## Introduction

Bananas (*Musa* spp.) are a nutritional fruit that is consumed worldwide, and they are also one of the most important commercial crops in tropical and subtropical developing countries. During the life cycle of banana plants, certain tissues accumulate a large amount of anthocyanin at a particular time (Horry and Jay, 1988). Anthocyanins play important roles in the growth and development of plants, such as adapting to biotic and abiotic stresses (Dixon and Paiva, 1995) and attracting pollinators, as well as in seed dispersal (Gould, 2004). In addition, anthocyanins are responsible for various colors and are also considered as nutrients beneficial for human health, which greatly influences the commodity value of plants (Tsuda, 2012; Wei et al., 2015).

In preliminary studies, the variety (Kitdamrongsont et al., 2008), content (Javed et al., 2001; Pazmiño-Durán et al., 2001), and function (Roobha et al., 2011) of banana anthocyanins have all been adequately determined, but the molecular mechanisms underlying anthocyanin biosynthesis and its regulation have only recently become recognized (Deng et al., 2019). Additionally, the gene that is involved in anthocyanin biosynthesis in banana plants has not been previously characterized by genetic methods because of the low transgenic efficiency of bananas.

The anthocyanin biosynthesis pathway is conserved in higher plants and has been widely investigated in various plants (Hichri et al., 2011; Zhang et al., 2014b; Naing and Kim, 2018). It was previously determined that the MYB-bHLH-WD40 complex regulates the expression of anthocyanin structural genes (Koes et al., 2005). Among them, the MYB transcription factors (TFs), in particular, are potent transcriptional regulators, and their temporal and spatial expression patterns can lead to dramatic changes in the plant anthocyanin profile (Jin et al., 2016). The first plant MYB TF that was identified as an anthocyanin biosynthesis regulator was the *c1* gene in maize (Paz-Ares et al., 1987). Since then, many MYB TFs from diverse species have been identified, and most of them are activator MYBs that stimulate anthocyanin structural gene expression. However, only studies produced in the past 5 years have been remarkably focused on MYB repressors, and only recently have their prevalence and importance become apparent (Chen et al., 2019a; Ma and Constabel, 2019).

The repressor MYBs in the anthocyanin pathway can be divided into two distinct groups: R2R3-MYBs and R3-MYBs. Interestingly, most of these repressors belong to subgroup 4 R2R3-MYBs. Some examples are *AtMYB4* (for *Arabidopsis thaliana*) (Jin et al., 2000), *BrMYB4* (for *Brassica rapa*) (Zhang et al., 2014a), *MdMYB16* (for *Malus × domestica*) (Xu et al., 2017), *VvMYB4* (for *Vitis vinifera*) (Cavallini et al., 2015), *NtMYB2* (for *Narcissus tazetta*) (Anwar et al., 2018), *TrMYB133* (for *Trifolium repens*) (Albert, 2015), *PtrMYB194* and *PtrMYB165* (for *Populus trichocarpa*) (Ma et al., 2018), and *FaMYB1* (for *Fragaria × ananassa*) (Aharoni et al., 2001). R2R3-MYB repressors are highly conserved in the N-terminal region, and most contain the conserved motif of [D/E]Lx2[R/K]x3Lx6Lx3R for interaction with bHLH proteins. By contrast, the protein sequences are divergent at the C-terminus, and the repressive function of these proteins usually depends on the distinct motifs in the C-terminal region (Zimmermann et al., 2004). Two conserved motifs are commonly found in most R2R3-MYB repressors. The first one, containing the core sequence pdLNLD/ELxiG/S, was referred to as an EAR motif (ethylene-responsive element binding factor–associated amphiphilic repression motif). For example, MdMYB16 requires the EAR motif to repress the expression of target genes, whereas MdMYB16, which lacks the EAR motif, loses its ability to inhibit the accumulation of anthocyanin in apple callus (Xu et al., 2017). The EAR motif acts as a negative regulator that participates in various physiological processes in plants by recruiting corepressors and may be involved in chromatin remodeling (Kagale and Rozwadowski, 2011). Another repression motif called the TLLLFR motif can also be found in some R2R3-MYB repressors, such as PtrMYB194 and PtrMYB165 (Ma et al., 2018). In the banana (*Musa acuminata* var. DH-Pahang) genome, 298 R2R3-MYB genes have been identified (D’Hont et al., 2012). Compared to model plants and other horticultural crops, there has been insufficient functional identification of R2R3-MYB repressors in banana. Only one R2R3-MYB repressor, namely, *MaMYB3*, has been well studied in banana, but it is unrelated to the regulation of anthocyanin biosynthesis (Fan et al., 2018).

To date, opposite and corresponding relationships between R2R3-MYB repressor expression and anthocyanin accumulation have been found in plants (Chen et al., 2019b). In *Vitis vinifera* cultivar Carmenere, the expression pattern of *VvMYB4-like* was the opposite to that of anthocyanin biosynthesis to prevent ectopic accumulation of anthocyanins (Pérez-Díaz et al., 2016). By contrast, *CsMYB3* expression was correlated with the accumulation of anthocyanins to provide feedback regulation and maintain anthocyanin homeostasis in citrus (Huang et al., 2020). Thus, it is difficult for us to determine whether the differentially expressed MYB is an activator or repressor solely through the relationship between gene expression and anthocyanin accumulation. Although Deng et al. (2019) first identified many differentially expressed MYBs that correlate with anthocyanin biosynthesis in banana by transcriptomics, it is still difficult for us to determine whether differentially expressed MYB is an activator or a repressor.

To determine the function of R2R3-MYB repressors in plants in repressing anthocyanin biosynthesis, genetic methods are usually used (Zhou et al., 2016). However, up to now, the MYB that is involved in anthocyanin biosynthesis in banana has not been previously characterized by genetic methods. Here, we characterized *MaMYB4* as a R2R3-MYB repressor that negatively regulates the accumulation of anthocyanin in banana leaf, which will be helpful for manipulating anthocyanin coloration in banana plants in the future.

## Materials and Methods

### Plant Materials and Growth Conditions

The embryogenic cell suspensions (ECSs) that were used for transformation were induced from the male flower of *Musa* spp. “Cavendish,” from the AAA group maintained in the national banana germplasm repository, Institute of Fruit Tree Research, Guangdong Academy of Agricultural Sciences. Plantlets were grown in a growth chamber under 30°C/28°C (day/night), a photon flux density of 300 μmol m^–2^ s^–1^ throughout a 12-h photoperiod, and a relative humidity of 60–80%. Eight-leaf-stage plantlets were used in the experiment. The leaves from three plants were cut into pieces (1.5 cm × 1.5 cm) and mixed well. Aliquots of the mixed tissues were frozen in liquid N_2_ and stored at −80°C until use. All samples were collected in triplicate.

### Vector Construction and Transformation

The full-length *MaMYB4* homolog open reading frame was amplified with the following primer set: forward (5′-ATGAG GAGCCCTTGTTGTGATAAG-3′) and reverse (5′-TTAGC GAAAGAGAAGGAGCGTCG-3′) and then introduced into the pMD18-T vector for sequencing. The right *MaMYB4* open reading frame fragment was collected and subcloned into the pBI121 vector after a *Sac*I and *Xba*I double digest. The plasmid pBI121: *MaMYB4* was digested with *Eco*RI and *Hin*dIII and then cloned into the pCAMBIA1301 vector (CAMBIA). The obtained pCMABIA1301:*MaMYB4* construct was transformed into *Agrobacterium tumefaciens* strain EHA105 using a previously described method (Hu et al., 2013). Briefly, the Cavendish banana ECS was cocultured with *A. tumefaciens* for 24 h at 26°C with gentle shaking in the dark. The ECSs were collected by removing supernatants, washing twice with sterile water, and culturing in proliferation medium supplemented with 25 mg/L hygromycin B and 200 mg/L cefotaxime at 26°C with shaking at 110 revolutions/min (rpm) in the dark. After this, the ECSs were cultivated in regenerated medium and subcultured every 3 weeks for three times until resistant mature somatic embryos appeared.

### Identification of Transgenic Plants

The genomic DNA, respectively, from samples including non-transformed controls and hygromycin-resistant plantlets was isolated by the improved CTAB method of Hu et al. (2013). Hygromycin-resistant plantlets were selected to be identified by polymerase chain reaction (PCR) using a pair of specific primers corresponding to 844-bp fragment of the *gus* coding region (forward 5′-GACGATTGCGTCGCATCGAC-3′ and reverse 5′-CGAAAAGTTCGACAGCGTCTCC-3′). Only those yielding the expected PCR fragments by the above primers were chosen for the Southern blot analyses. Four independent transgenic plants were obtained, named MaMYB4OE, MaMYB4-2OE, MaMYB4-3OE, and MaMYB4-4OE. Southern blot analyses were done as we previously described with minor modified (Dou et al., 2019). Total genomic DNA was isolated from young leaf tissues (2.0 g) of selected plantlets using plant DNA isolation kit (TAKARA, Japan). Purified DNA was digested with *Eco*RV and fractionated on 1.0% agarose gel. DNA fragments containing the 303-bp fragment of the third exon of *MaMYB4* gene were amplified from the pCMABIA1301:*MaMYB4* construct with the forward primer (5′- AGAAGCTCACAAGCATG-3′) and reverse primer (5′-AGCGAAAGAGAAGGAGC-3′), and used as a hybridization probe and labeled with digoxigenin. DNA of wild-type plantlets used as positive control and empty plasmid pCAMBIA1301 was digested with *Bam*HI used as negative control. Labeling, hybridization, and washing were carried out using the DIG labeling and Luminescent Detection Kits (Roche, Switzerland). Chemiluminescence was detected on the nylon filters using a chemiluminescence enabled gel-documentation system. Because of MaMYB4OE and MaMYB4-2OE accumulated the least anthocyanin content, they were chosen for subsequent quantitative analysis, and MaMYB4OE was used for subsequent qualitative analysis.

### Sequence Alignment and Phylogenetic Analysis

The full-length amino acid sequences of MaMYB4 from banana and R2R3-MYB repressors from other plants were aligned using ClustalW software. A phylogenetic tree was constructed using MEGA 5.05 software (Tamura et al., 2011). Node support was estimated using a neighbor-joining bootstrap analysis (1,000 bootstrap replicates).

### Subcellular Localization

To determine the subcellular localization of MaMYB4, the *MaMYB4* gene was amplified by PCR and cloned into the pYL322-d1 vector containing a red fluorescent protein (mCherry) reporter gene (digested with *Eco*RI in advance) to produce the fusion construct pYL322-MaMYB4-mCherry under control of the *cauliflower mosaic virus* 35S promoter by using a One Step Cloning Kit (Vazyme Biotech Co., Ltd., Nanjing, China). The fusion construct and the control vector (pYL322-ICE1-GFP) were transferred into rice protoplasts (Marx, 1987). The transformed protoplasts were visualized under a universal fluorescence microscope (Olympus BX61, Tokyo, Japan).

### Anthocyanin Extraction and Measurement

The anthocyanin content was determined based on a previously reported protocol (Mita et al., 1997) with modifications. Plantlets of *MaMYB4-*overexpressed bananas and wild-type were collected and weighed, after which anthocyanins were extracted in 2 mL of buffer A (50 mM KCL and 150 mM HCL, pH 1.0) and 2 mL of buffer B (400 mM sodium acetate and 240 mM HCl, pH 4.5), respectively, for 30 min at 4°C with ultrasonic vibration. The extracts were then centrifuged for 10 min at 12,000 rpm. The resulting supernatant (2 mL) was used to measure the A510 values, and (A510 at pH 1.0 - A510 at pH 4.5) × 484/24825/fresh weight × 100 was considered as the anthocyanin content. All experiments were independently repeated six times.

### RNA Extraction and Reverse Transcription–Quantitative Polymerase Chain Reaction Analysis

Total RNA was extracted from different tissues using a plant RNA isolation kit (Column Plant RNAOUT 2.0, TIANDZ, China) according to the manual’s instructions. For each sample, the first-strand cDNA was synthesized according to the Prime Script^TM^ reverse transcription–PCR (RT-PCR) kit protocol (Takara, Japan). Primer pairs for RT–real-time quantitative PCR (RT-qPCR) (see Supplementary Data 1) were designed using Primer Premier 5.0 software (Premier Biosoft, Palo Alto, CA, United States). PCR reactions were performed on a LightCycler^®^ 480 II with LightCycler480 Service Software (Roche, Germany) by using the LightCycler^®^ Premix EX Taq (Perfect Real Time) kit protocol (Takara, Japan). The efficiency of primers for the target gene and internal control gene (*25S ribosomal RNA*) was assessed. No-template controls were also set for each primer pair as a blank control. All experiments were independently repeated three times.

### Yeast Two-Hybrid Analysis

The yeast two-hybrid assay was performed using the Matchmaker Gold Yeast Two-Hybrid Systems (Clontech). The coding sequences of *bHLH* were cloned in-frame with the *GAL4* DNA-binding domain sequence in the bait vector pGBKT7. Next, we fused the coding sequence of *MaMYB4* in-frame with the sequence of the *GAL4* activation domain in the prey vector pGADT7. The bait plasmid pGBKT7-bHLH and the prey plasmid pGADT7-MaMYB4 were cotransformed into yeast strain AH109 by the lithium acetate method and grown on DDO medium (minimal media double dropouts, SD medium with -Leu/-Trp) for 3 days according to the manufacturer’s protocol. Transformed colonies were plated onto QDO medium (minimal media quadruple dropouts, SD medium with -Leu/-Trp/-Ade/-His), and the possible interaction between MaMYB4 and MabHLH was evaluated according to their growth status. The bait plasmid pGBKT7-ICE1 and the prey plasmid pGADT7-MAPK3 were used as a positive control. Additionally, the bait plasmid pGBKT7-ICE1 and the prey plasmid pGADT7-MAPK9 were used as a negative control.

### Dual-Luciferase Transient Expression Assay

For the analysis of transrepression of *MaCHS*, *MaDFR*, *MaANS*, and *MabHLH* promoter activity by MaMYB4, transient transcription dual-LUC assays using ECSs of banana were performed. The protoplast isolation and polyethylene glycol–mediated transformation were carried out as previously described (He et al., 2018). We isolated protoplasts from 5-day-old subcultured ECSs of *Musa* spp. “Cavendish,” AAA group. Next, a total of 20 μg DNA (effector constructs 35S:*MaMYB4*-GFP and pGreen-CHSpro:LUC reporter or pGreen-DFRpro:LUC reporter, pGreen-ANSpro:LUC reporter, and pGreen-bHLHpro:LUC reporter) was transfected into protoplasts and incubated overnight. The luciferase activity was measured using a luminometer (GloMax 20/20; Promega, Madison, WI, United States) with the Dual-Luciferase Reporter Assay System (E1910, Promega), according to the manufacturer’s instructions. All experiments were independently repeated six times.

### RNA Sequencing and Sequence Analysis

Total RNA was isolated from the leaves of eight-leaf-stage plantlets of *MaMYB4-*overexpressed plants and wild-type plants in three biological replicates as previously described (Yang et al., 2015). RNA samples were treated with DNase before being quality checked using an Agilent 2100 Bioanalyzer. Total RNA was prepared for a strand-specific TruSeq^TM^ RNA-Seq library, and all six samples were sequenced over three lanes on an Illumina HiSeq 4000, with 150-bp paired-end reads. Differential expression analysis was performed using the DEGSeq R package 1.12.0. *P* values were adjusted using the Benjamini and Hochberg method. A corrected *P* value of 0.05 and log_2_ (fold change) of 1 were set as the threshold for significantly differential expression. Gene ontology enrichment analysis of differentially expressed genes was implemented by the GOseq R package, in which gene length bias was corrected. Gene ontology terms with a corrected *P* value less than 0.05 were considered significantly enriched by differentially expressed genes. All sequences generated in this study have been deposited in the National Center for Biotechnology Information Sequence Read Archive^1^ with project number PRJNA662185.

### Metabolome Analysis

For sample preparation and extraction, each freeze-dried leaf was crushed using a mixer mill (MM 400, Retsch) with a zirconia bead for 1.5 min at 30 Hz. Next, 100 mg of powdered leaf was extracted overnight at 4°C with 1.0 mL 70% aqueous methanol. Following centrifugation at 10,000 × *g* for 10 min, the extracts were absorbed (CNWBOND Carbon-GCB SPE Cartridge, 250 mg, 3 mL) and filtered (SCAA-104, 0.22-μm pore size) before liquid chromatography–mass spectrometry (LC-MS) analysis.

The sample extracts were analyzed using an LC–electrospray ionization–tandem MS (ESI-MS/MS) system, high-performance LC, Shim-pack ultrafast LC Shimadzu CBM30A system, quadrupole-linear ion trap hybrid mass spectrometer (Applied Biosystems 4500 QTRAP). The analytical conditions were as follows: high-performance LC: column, Waters Acquity ultrahigh performance LC HSS T3 C18 (1.8 μm, 2.1 mm × 100 mm); solvent system, water (0.04% acetic acid): acetonitrile (0.04% acetic acid); gradient program, 100:0 vol/vol at 0 min, 5:95 vol/vol at 11.0 min, 5:95 vol/vol at 12.0 min, 95:5 vol/vol at 12.1 min, 95:5 vol/vol at 15.0 min; flow rate, 0.40 mL/min; temperature, 40°C; injection volume: 5 μL. The effluent was alternatively connected to an ESI-triple quadrupole-linear ion trap mass spectrometer.

Linear ion trap and triple quadrupole scans were acquired on a triple quadrupole–linear ion trap mass spectrometer, API 4500 QTRAP LC/MS/MS system, equipped with an ESI turbo ion-spray interface, operating in a positive ion mode and controlled by Analyst 1.6.3 software (AB Sciex). The ESI source operation parameters were as follows: ion source, turbo spray; source temperature 550°C; ion spray voltage 5,500 V; ion source gas I, gas II; and curtain gas were set at 55, 60, and 25.0 psi, respectively; the collision gas was high. Instrument tuning and mass calibration were performed with 10 and 100 μmol/L polypropylene glycol solutions in triple quadrupole and linear ion trap modes, respectively. Triple quadrupole scans were acquired as multiple reaction monitoring (MRM) experiments with collision gas (nitrogen) set to 5 psi. Declustering potential and collision energy for individual MRM transitions were performed with further declustering potential and collision energy optimization. A specific set of MRM transitions was monitored for each period according to the metabolites eluted within this period. All samples were independently collected and tested in triplicate.

### Statistical Analysis

Each experiment contained a minimum of six plants per treatment and was repeated three or six times. The data were recorded as the mean ± standard errors of three or six independent biological replicates. Statistical differences between samples were analyzed by Student *t* test (*P* < 0.05 or 0.01).

## Results

### Identification, Expression Pattern, and Subcellular Localization of *MaMYB4*

Based on the banana genome^2^ and the MYB conserved domain (PF00249) from HMMER^3^, we identified 298 R2R3-MYBs. Among them, 21 R2R3-MYBs were found that contained the EAR motif, and four of them also contained the TLLLRF motif, namely, Ma06_g11140.1, Ma03_g21920.1, Ma08_g16760.1, and Ma10_g19970.1, and they all belong to subgroup 4 according to the classification of MYB in *A. thaliana* (Supplementary Figure 1). Among them, *MaMYB3* (Ma06_g11140.1) has been proven to be related to decreased degradation of starch and delayed fruit ripening in banana, but it is unrelated to the regulation of anthocyanin (Fan et al., 2018). Thus, we subsequently chose Ma03_g21920.1, which was the most homologous with *AtMYB4*, for further research and refer to it as *MaMYB4* hereafter.

**FIGURE 1:**
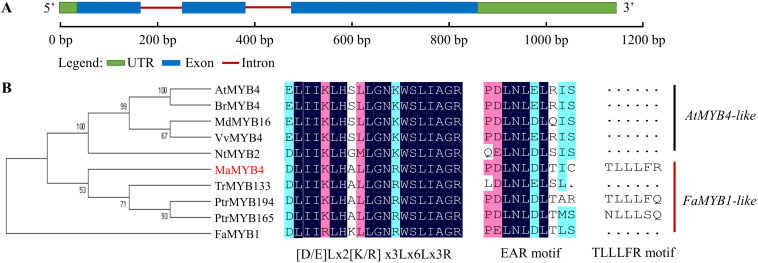
Sequence analysis of *MaMYB4* and other nine R2R3-MYB repressor transcription factors (TFs). **(A)** Schematic representation of the sequence organization of *MaMYB4*. **(B)** Phylogenetic analysis and amino acid sequence alignment of MaMYB4 and other nine R2R3-MYB repressors from various organisms. The amino acid sequences were aligned using the Clustal Omega, and the tree was drawn with neighbor joining method using MEGA software 5.0. They have the conserved motif of [D/E]Lx2[R/K]x3Lx6Lx3R and EAR. In addition, MaMYB4 contains TLLLFR motif, and it belongs to FaMYB1-like R2R3-MYB repressor (Chen et al., 2019b).

The cDNA sequence of *MaMYB4* was 1142 bp in length, including a complete open reading frame of 820 bp with a 5′-untranslated region of 36 bp and 3′-untranslated region of 286 bp, containing 3 exons and 2 introns and encoding a protein of 213 amino acids with a predicted molecular mass of 24.1 kDa (Figure 1A). Sequence analysis revealed that the N-terminal region includes a C1 motif (LlsrGIDPxT/SHRxI/L) and an R2R3 domain, and a C2 (pdLNLD/ELxiG/S) and C5 (TLLLFR) motif in the C-terminal region (Supplementary Figure 2A). To better understand the function of *MaMYB4*, a phylogenetic analysis with known anthocyanin-related R2R3-MYB repressors was performed (Figure 1B). MaMYB4 was clustered into the *FaMYB1-like* clade, which was found to act upon bHLH factors and interfere with the proper assembly of the MYB-bHLH-WD40 complex to repress anthocyanin biosynthesis (Albert et al., 2014). Moreover, MaMYB4 has the conserved motif of [D/E]Lx2[R/K]x3Lx6Lx3R within the R3 domain for interaction with the bHLH. Both the phylogenetic and sequence analyses indicated that *MaMYB4* may be a novel R2R3 MYB repressor in banana.

To determine the relationship between *MaMYB4* expression and anthocyanin accumulation in banana, the expression pattern of *MaMYB4* in the purple part and green part of one banana leaf was investigated. RT-qPCR analyses revealed that *MaMYB4* was highly expressed in the anthocyanin-accumulating part of banana leaves (Supplementary Figures 2B,C). This indicated that *MaMYB4* is expressed when anthocyanins are being biosynthesized. Based on these data, we hypothesized that *MaMYB4*, as a repressor, provides feedback regulation of anthocyanin accumulation in banana.

**FIGURE 2:**
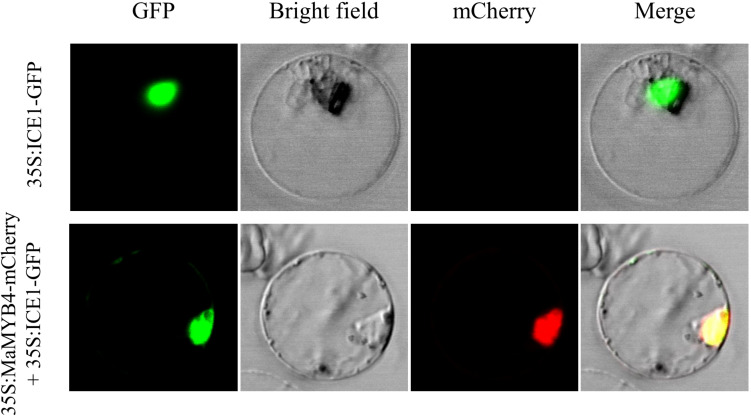
Subcellular localization analysis of MaMYB4. The open reading frames of MaMYB4 and ICE1 (used as the nucleus marker) were in framed with GFP and mCherry N-terminus, respectively. The obtained constructs driven by 35S promoter were transient expressed in rice protoplasts and visualized by fluorescence microscopy. The GFP fluorescence signals indicated the localization of nucleus. Overlay images show colocalization of GFP and mCherry signals.

To identify the subcellular localization of MaMYB4 protein, the open reading frame of *MaMYB4* was in-frame with the N-terminus of mCherry protein, and the ICE1-GFP protein, which is known to be localized in the nucleus in banana, was used as a positive control in this study. The subcellular localization was determined by transient expression in a rice protoplast system. As shown in Figure 2, the red fluorescence generated by MaMYB4-mCherry was distributed in the same area as the green fluorescence generated by ICE1-GFP, which suggested that *MaMYB4* is a nuclear-localized TF.

### Overexpression of *MaMYB4* in Banana Led to a Decrease in Anthocyanins

**FIGURE 3:**
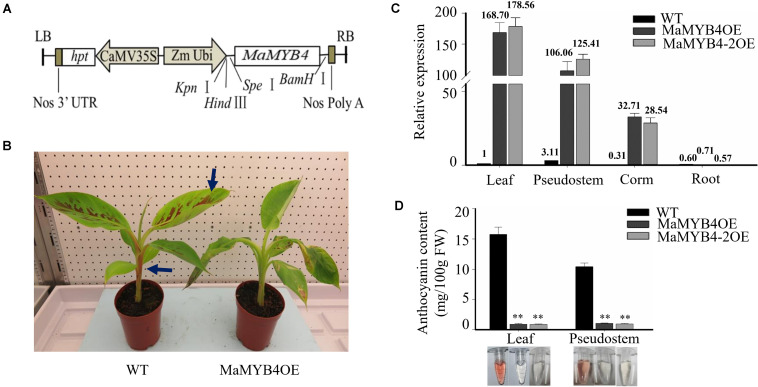
Analysis of *MaMYB4* overexpressors. **(A)** Schematic diagrams of the constructs used for *Agrobacterium*-mediated transformation. **(B)** Phenotypic characterizations of wild type and MaMYB4OE following 1.5 months for cultivation in incubator. Expression level of *MaMYB4* **(C)** and anthocyanin content **(D)** in different tissue of wild type and two *MaMYB4* overexpressors. Asterisks on the bar indicate significant difference according to Student *t* test (*P* < 0.05). Bar indicates standard deviation (*n* = 6).

To investigate the role of *MaMYB4* in the regulation of anthocyanin biosynthesis in banana, the cDNA of *MaMYB4* was overexpressed in wild-type plants under control of constitutive *ZmUBI* promoter (Figure 3A). Constructs for overexpression were used to transform diploid banana ECS via *Agrobacterium*-mediated transformation. Stable integration and overexpression of *MaMYB4* in transgenic banana plants were proved by Southern blot analysis, and there were 2, 3, 1, and 1 copies T-DNAs in the MaMYB4OE, MaMYB4-2OE, MaMYB4-3OE, and MaMYB4-4OE, respectively (Supplementary Figure 3). The *MaMYB4-*overexpressed plants and wild-type plants showed no remarkable phenotypic difference before the wild-type began to accumulate anthocyanins, excluding the possibility that *MaMYB4* functions as a positive regulator of anthocyanin biosynthesis. After 1.5 months of cultivation in an incubator, the leaves and pseudostems of the wild type changed from green to purple, whereas those of the transgenic plants remained green (Figure 3B). In order to verify that this phenotypic difference was caused by *MaMYB4* and related to anthocyanin, the expression pattern of *MaMYB4* and the anthocyanin content were determined in the MaMYB4OE, MaMYB4-2OE, and wild type. In two transgenic plants, the transcript of *MaMYB4* was significantly higher than that of wild type, especially in leaves and pseudostems (Figure 3C). Moreover, the anthocyanin content of leaves and pseudostems in MaMYB4OE plants was 0.91 and 1.04 mg/100 g fresh weight (FW), respectively, which was significantly less than that of the wild type (15.75 and 10.42 mg/100 g FW, respectively) (Figure 3D), suggesting the important role of *MaMYB4* in negatively regulating the biosynthesis of anthocyanin in banana plants.

**FIGURE 4:**
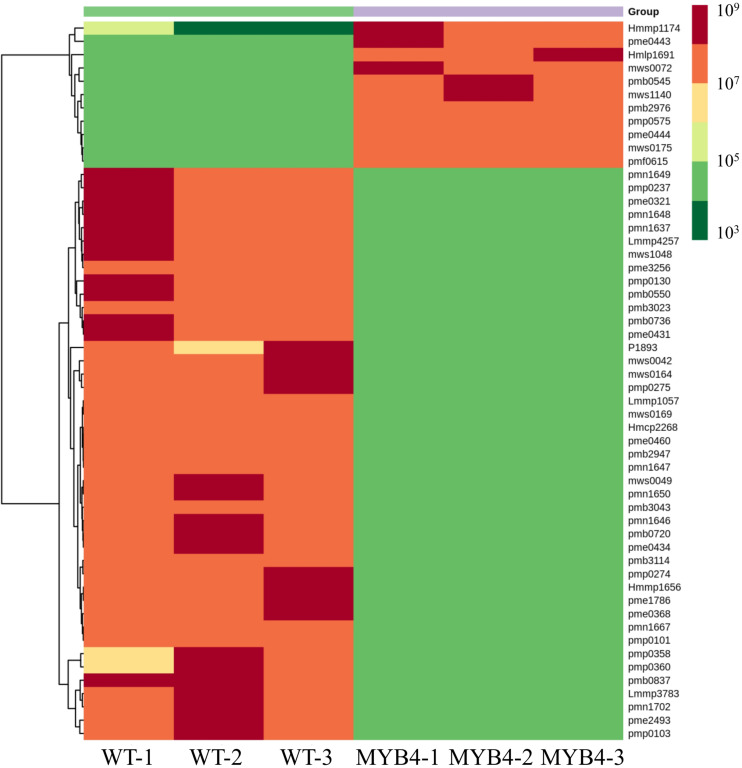
The heat map of the differently accumulated flavonoids in MaMYB4OE plants by metabolomics.

*MaMYB4* overexpressors exhibited a stronger repression phenotype in anthocyanin accumulation. To further identify the effect of *MaMYB4* overexpression on flavonoids, metabolomics profiling was performed with the leaves of wild-type and *MaMYB4*-overexpressed plants as materials. A total of 195 flavonoids were identified. Among them, 54 differentially expressed flavonoids (DEFs) were identified, with 39 DEFs exclusively in wild type, 10 DEFs in *MaMYB4* overexpressors only, and 5 DEFs in both. Compared to the wild type, the number of flavonoids that were decreased in abundance (43) was three times more than that which was increased in abundance (11) in the leaves of *MaMYB4*-overexpressed plants (Figure 4). Interestingly, the 43 DEFs with decreased abundance involved all major flavonoids, such as flavonols, anthocyanins, and proanthocyanidins. Moreover, there was a decrease in all three identified cyanidins (cyanidin 3-O-glucoside, cyanidin 3-rutinoside, and cyanidin 3-O-galactoside) and the delphinidin (delphinidin 3-O-rutinoside) that is considered to be the main anthocyanin that determines the color of banana leaves (Supplementary Data 2). Based on these data, we hypothesized that overexpression *MaMYB4* in banana led to a decrease in anthocyanins and a subsequent inability in the leaves to turn purple.

### Overexpression of *MaMYB4* Downregulated the Transcription of Anthocyanin Synthesis-Associated Genes

**FIGURE 5:**
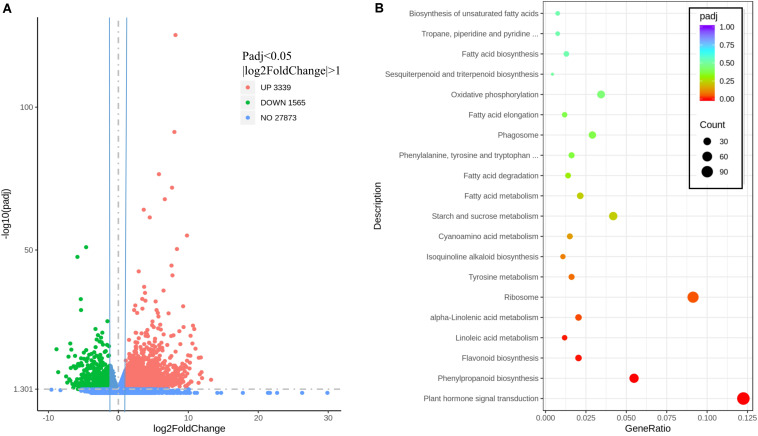
The volcano map and enriched pathway represented by the genes affected by MaMYB4. **(A)** Identification of the differentially expressed genes. The DEGs were filtered for *P* < 0.05 and | log_2_FoldChange| > 1, and 3,339, 1,565, and 27,873 genes were upregulated, downregulated, and not differentially changed in the MaMYB4OE banana leaves, respectively. **(B)** KEGG analysis of the 20 most enriched pathways. The coloring of the *q* values represents the significance of the enrichment factor. A circle shows the target genes that are involved, and the size is proportional to the gene number. The *y*-axis shows the name of the enrichment pathway. The *x*-axis indicates the enrichment factor.

To investigate the effect of *MaMYB4* overexpression on the entire transcriptome, RNA-Seq profiling was performed with the leaves of wild-type and *MaMYB4* overexpressors as materials. The fragments per kilobase of transcript per million fragments mapped values are presented from three biological replicates for each sample (Supplementary Data 3). At a threshold of a twofold expression ratio and a *q* < 0.05, 4904 genes were differentially expressed in *MaMYB4* overexpressors compared to the wild type. Among the differentially expressed genes, 1,565 genes were downregulated, and 3,339 genes were upregulated (Figure 5A). To determine the biological interpretation of differentially expressed genes, a Kyoto Encyclopedia of Genes and Genomes pathway analysis was performed. The results showed that 931 genes were assigned to the top 20 enriched pathways. The first three significantly enriched terms were “plant hormone signal transduction,” “phenylpropanoid biosynthesis,” and “flavonoid biosynthesis” according to *P* value (Figure 5B), and 114, 51, and 19 related genes were enriched, respectively. Interestingly, the number of downregulated genes (15) was threefold greater than that upregulated (4) in the “flavonoid biosynthesis” class, whereas it was half in the “phenylpropanoid biosynthesis” class. In addition, all four upregulated genes in the “flavonoid biosynthesis” class were enriched in the upstream of anthocyanin synthesis, which has little effect on anthocyanin synthesis.

Because the reduction of anthocyanin may be the result of the differential expression of anthocyanin biosynthesis-related genes, we studied them further. In total, 10 DEGs, including almost all types of the key enzyme-coding structural genes that participate in anthocyanin biosynthesis, were annotated according to homologous genes in *Arabidopsis*. The DEGs consisted of two chalcone synthase (*CHS*), two chalcone isomerase (*CHI*), one flavonoid 3′-monooxygenase (*F3’H*), two dihydroflavonol 4-reductase (*DFR*), one leucoanthocyanidin dioxygenase/anthocyanidin synthase (*LDOX/ANS*), and two anthocyanidin 3-O-glucosyltransferase (*UFGT*) genes (Table 1). Moreover, all of those genes were downregulated in *MaMYB4* overexpressors.

**TABLE 1:** Expression of anthocyanin synthesis-related genes in the transcriptome data (*MYB4*-overexpressors vs. wild-type).

Homologous *Arabidopsis* Gene	Locus ID of Homolog	Log2FC	Type
AtCHS (AT5G13930)	Ma06_g12370.1	–7.31	Downregulated
	Ma06_g17870.1	–1.53	Downregulated
AtCHI/TT5 (AT3G55120)	Ma04_g17380.1	–4.94	Downregulated
	Ma11_g21950.1	–6.72	Downregulated
AtF3′H/TT7 (AT5G07990)	Ma03_g06970.1	–1.71	Downregulated
AtDFR/TT3 (AT5G42800)	Ma03_g32330.1	–4.34	Downregulated
	Ma04_g10620.1	–7.13	Downregulated
AtANS/AtLDOX (AT4G22880)	Ma05_g03850.1	–6.40	Downregulated
AtUFGT1 (AT5G17050)	Ma01_g07150.1	–1.71	Downregulated
	Ma01_g07170.1	–1.70	Downregulated
AtTT8/AtbHLH42 (AT4G09820)	Ma03_g18660.1	–4.16	Downregulated
	Ma06_g24180.1	–4.12	Downregulated
AtMYB4 (AT4G38620)	Ma08_g16760.1	–4.91	Downregulated
	Ma10_g19970.1	–1.581	Downregulated
AtMYB75/PAP1	Ma03_g07850.1	–1.85	Downregulated
	Ma09_g15440.1	–5.13	Downregulated
	Ma10_g17650.1	–3.87	Downregulated

**FIGURE 6:**
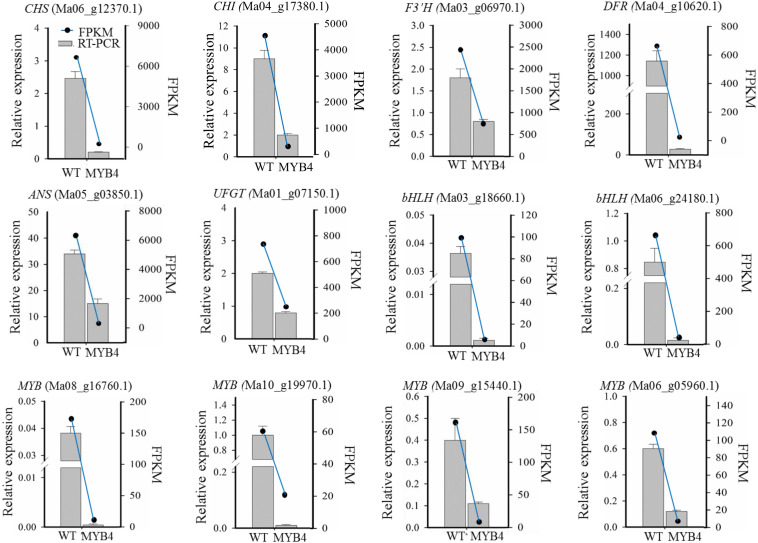
Gene expression changes as assessed by RNA-Seq and RT-qPCR. The labels in parentheses were representative genes involved in anthocyanin synthesis. The relative expression, detected by RT-qPCR, was represented by a bar graph, and the left ordinate indicates the expression level. FPKM of gene, according to RNA-Seq data, was represented by a line graph, and the right ordinate indicates the FPKM level.

Among the DEGs, we also found that many TFs were downregulated, such as three *MYB* (Ma03_g07850.1, Ma09_g15440.1, and Ma10_g17650.1) and two *bHLH* genes (Ma03_g18660.1 and Ma06_g24180.1), which were homologous to the member of the MYB-bHLH-WD40 complex in *Arabidopsis*. Unexpectedly, the two R2R3-MYBs (Ma08_g16760.1 and Ma10_g19970.1) with EAR and TLLLFR motifs were downregulated in *MaMYB4* overexpressors (Table 1). Reduced expression was observed in 10 anthocyanin structural genes and five regulation TFs in the *MaMYB4* overexpressors, and this corresponded to the broad reduction in accumulation of anthocyanin in these plants. To test the reliability of transcriptome data, six anthocyanin structural genes and six regulation TFs that were differentially expressed in *MaMYB4* overexpressors were chosen for the RT-qPCR assay. Gene expression as elucidated by RT-qPCR exhibited similar trends with that of the transcriptome data, with some variation in the magnitude (Figure 6). This result clearly suggested that *MaMYB4* overexpression had directly or indirectly affected genes on the anthocyanin synthesis pathway.

**FIGURE 7:**
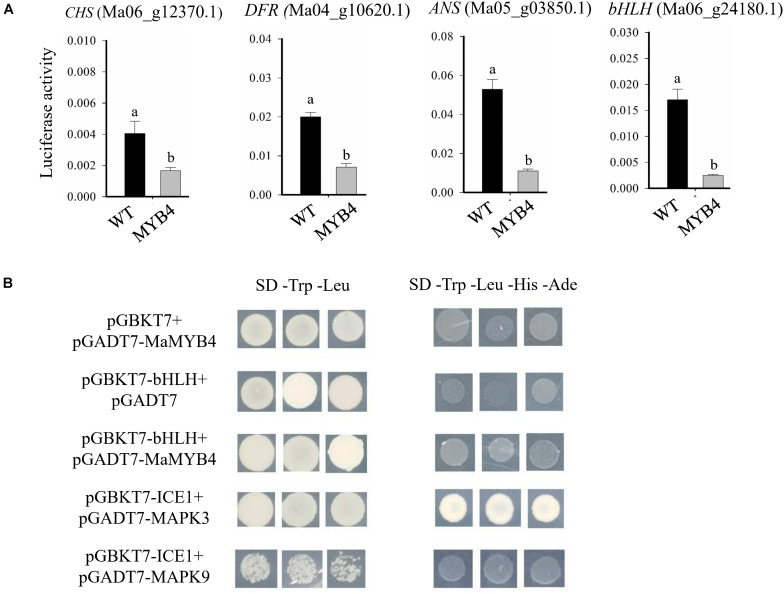
MaMYB4 did not physically interact with bHLH, but inhibited the transcriptional activity of *CHS*, *DFR*, *ANS*, and *bHLH* promoters. **(A)** The promoter activity assays in the transient expression study revealed that MaMYB4 could repress the activity of *CHS*, *DFR*, *ANS*, and *bHLH* promoter. We used a dual-LUC reporter plasmid encoding the firefly luciferase (LUC) gene driven by the *CHS*, *DFR*, *ANS*, or *bHLH* promoter and a Renilla luciferase (REN) gene driven by the constitutive *cauliflower mosaic virus* (CaMV) 35S. We transiently expressed the pGreen-*CHS*pro:LUC, pGreen-*DFR*pro:LUC, pGreen-*ANS*pro:LUC, and pGreen-*bHLH*pro:LUC in banana protoplasts together with either 35S:MaMYB4-GFP or 35S:GFP. Different lowercase letters indicate the significant differences among treatments by Student *t* test at *P* < 0.001. **(B)** The yeast two-hybrid assay showing no interaction between MaMYB4 and bHLH. The bait plasmid pGBKT7-ICE1 and the prey plasmid pGADT7-MAPK3 were used as positive control. Meanwhile, the bait plasmid pGBKT7-ICE1 and the prey plasmid pGADT7-MAPK9 were used as negative control.

To clarify the mechanism of the opposite expression patterns between *MaMYB4* and anthocyanin synthesis–associated genes, we analyzed the transcriptional *cis-*elements of the above 10 anthocyanin structural genes and two bHLH promoters. Those promoter sequences were cloned from banana (*Musa* spp. “Cavendish,” AAA group), and they contained at least one conserved MYB-recognition element, suggesting that *MaMYB4* can bind to their promoter. We then analyzed whether MaMYB4 affected the transcriptional activity of four representative genes on its target promoters using a transient transcription assay in banana protoplasts. The LUC activity level from the pGreen-CHSpro:LUC, pGreen-DFRpro:LUC, pGreen-ANSpro:LUC, and pGreen-bHLHpro:LUC reporter was 2. 4-, 2. 8-, 4. 8-, and 6.7-fold lower, respectively, when we coexpressed *MaMYB4* in the protoplasts relative to coexpressed *GFP* (Figure 7A). However, the R2R3-MYB repressor TF can incorporate into the MYB-bHLH-WD40 complex and regulate the expression of anthocyanin synthesis–associated genes. We tested the interaction between MaMYB4 and bHLH (Ma06_g24180.1) using a yeast two-hybrid assay. As shown in Figure 7B, MaMYB4 did not interact with bHLH in yeast cells. The results revealed that MaMYB4 did not physically interact with bHLH, but inhibited the transcriptional activity of anthocyanin synthesis–associated genes.

## Discussion

### *MaMYB4*, an R2R3-MYB Repressor Transcription Factor, Is Involved in the Regulation of Anthocyanin Biosynthesis in Banana

The MYB TFs comprise one of the largest families of transcriptional regulators in plants. Since the first MYB-promoting anthocyanin synthesis was discovered 30 years ago, it has been revealed that numerous MYB transcriptional activators are the major determinant regulators in the MYB-bHLH-WD40 complex in anthocyanin biosynthesis (Chen et al., 2019a). Recently, increasing evidence has suggested that anthocyanin biosynthesis is also controlled by MYB repressors. However, no data have been obtained in related studies on bananas (Ma and Constabel, 2019). MYB repressors usually possess some repressive motifs, such as the EAR (containing C1 and C2) motif and the TLLLFR (C5) motif, which are involved in the repression of genes in anthocyanin biosynthesis by complex regulatory mechanisms. *MaMYB4* was identified in banana based on this characteristic. Sequence analysis revealed that the MaMYB4 protein contained a conserved R2R3 domain and a C1 motif in the N-terminal region, and a C2 and C5 motif in the C-terminal region (Supplementary Figure 2). Moreover, multiple sequence alignment revealed that *MaMYB4* was clustered into the clade of the *FaMYB1-like* R2R3 repressor (Figure 1B). Therefore, it appears that the MaMYB4 is a novel R2R3 MYB repressor in banana.

Subcellular localization analyses suggested that MaMYB4 is a nuclear-localized TF. RT-qPCR analyses revealed that *MaMYB4* was activated when banana leaves accumulated a large amount of anthocyanin (Supplementary Figure 2). These results were similar to previous findings of many anthocyanin *FaMYB1-like* R2R3 repressors, such as *FaMYB1* (Aharoni et al., 2001), *PtrMYB57* (Wan et al., 2017), and *Tr-MYB133* (Albert et al., 2011). Furthermore, it was previously suggested that these R2R3-MYB repressors were expressed when anthocyanins were being biosynthesized and provided feedback regulation (Chen et al., 2019b), and we speculated that *MaMYB4* is also involved in the regulation of anthocyanin biosynthesis in banana.

### *MaMYB4* Represses the Regulation of Anthocyanin Accumulation in Banana

To determine how R2R3-MYB represses anthocyanin biosynthesis in plants, transient or stable transgenic methods are usually used (Xu et al., 2017; Anwar et al., 2018). Here, MaMYB4 overexpressors showed a stronger repression phenotype during anthocyanin accumulation. During the early culture stage, the MaMYB4-overexpressed banana and wild type showed no remarkable phenotypic difference, excluding the possibility that *MaMYB4* functions as a positive regulator of anthocyanin biosynthesis. After 1.5 months, the leaves and pseudostems of the wild type changed from green to purple; the anthocyanins accumulated up to 15.75 and 10.42 mg/100 g FW, respectively, which was significantly more than the contemporaneity of the transgenic plants (Figure 3). This change suggested that *MaMYB4* plays an important role in negatively regulating the biosynthesis of anthocyanin in banana.

To further identify the effect of *MaMYB4* overexpression on flavonoids, metabolomics profiling was performed with the leaves of wild-type and *MaMYB4*-overexpressed plants as materials. Compared to wild type, the number of decreased flavonoids (43) was three times more than increased (11) in the leaves of *MaMYB4* overexpressors (Figure 4). Interestingly, the 43 decreased DEFs involved all major flavonoids, such as flavonols, anthocyanins, and proanthocyanidins. Among them, all three cyanidins (cyanidin 3-O-glucoside, cyanidin 3-rutinoside, and cyanidin 3-O-galactoside) and one delphinidin (delphinidin 3-O-rutinoside) were decreased. In contrast, one of two pelargonins, as well as one petunidin and three malvidins that belong to pelargonin ramification, were significantly accumulated in *MaMYB4* overexpressors. According to previous studies, the purple pigments in banana leaves were mainly cyanidins and delphinidins, and formed the purple patches on banana leaves (Horry and Jay, 1988; Javed et al., 2001). Based on these data, we hypothesized that overexpression *MaMYB4* in banana led to a decrease in cyanidins and delphinidins in leaves, and this is an important reason why the leaves in *MaMYB4* overexpressors do not accumulate purple patches like those observed in wild-type leaves.

### *MaMYB4* Decreased Anthocyanin Accumulation by Downregulation of the Transcription of Anthocyanin Synthesis–Associated Genes

Several enzyme-coding structural genes (including *CHS*, *CHI*, *F3*′*H*, *DFR*, *ANS*, and *UFGT*) and TFs (including the MYB-bHLH-WD40 complex) have been found to promote anthocyanin biosynthesis, whereas R2R3-MYB repressors can prevent anthocyanin accumulation by acting upon MYB-bHLH-WD40 complex, restraining the abundance of bHLH and MYB activators, and directly binding to the promoter of enzyme-coding structural genes (Saito et al., 2013; Xu et al., 2015). In our study, *MaMYB4* overexpression caused a significant decrease in the accumulation of anthocyanin in the banana leaves (Figure 3). Our transcriptome data showed that 10 anthocyanin structural genes were downregulated in *MaMYB4* overexpressors according to homologous genes in *Arabidopsis*. Namely, two *CHS*, two *CHI*, one *F3’H*, two *DFR*, one *LDOX/ANS*, and two *UFGT* (Table 1).

In addition to enzyme-encoding genes, multiple transcriptional activators were repressed by *MaMYB4* overexpression, for example, three MYB (Ma03_g07850.1, Ma09_g15440.1, and Ma10_g17650.1) and two bHLH genes (Ma03_g18660.1 and Ma06_g24180.1) (Table 1). Subsequently, these results were confirmed to be reliable by RT-qPCR assay (Figure 6). Ten anthocyanin structural genes and five regulatory TFs exhibited reduced expression in the *MaMYB4* overexpressors, and this corresponded to the broad reduction in accumulation of anthocyanin in these plants.

Recent studies found that *FaMYB1-like*–type repressors inhibited anthocyanin biosynthesis mainly at three levels. First, they are able to interfere with the proper assembly of the MYB-bHLH-WD40 activation complex by bridging through their bHLH factors, resulting in the repression of transcription of late anthocyanin structural genes, such *ANS*, *DFR*, and *UFGT*. Second, *FaMYB1-like*–type repressors restrain the abundance of bHLH and MYB activators. Third, they inhibit the expression of anthocyanin structural genes (Albert et al., 2014). In our study, 10 anthocyanin structural genes, including almost all types of key enzyme-coding structural genes, were downregulated when *MaMYB4* was overexpressed in banana. As mentioned above, both *FaMYB1-like* types repress the modes mentioned above. The conserved motif of [D/E]Lx2[R/K]x3Lx6Lx3R for interaction with bHLH was identified within the R3 domain in MaMYB4 protein (Figure 1B). We hypothesized that there is a possibility of interaction between MaMYB4 and MWB. Unexpectedly, a yeast two-hybrid analysis confirmed that they did not interact with each other (Figure 7B). However, *FaMYB1-like*–type repressor can directly bind to the promoter of structural genes and regulate TFs (Chen et al., 2019b).

In our study, three MYB genes and two bHLH genes, which were highly homologous to *AtMYB75/PAP1* and *AtTT8*, respectively, were both repressed by *MaMYB4* (Table 1 and Figure 6), and it was previously suggested that *AtMYB75/PAP1* and *AtTT8* were the major determinant regulators in the MYB-bHLH-WD40 complex in anthocyanin biosynthesis (Zuluaga et al., 2008). Consistent with this, *MaMYB4* strongly inhibited the *CHS* (Ma06_g12370.1), *DFR* (Ma04_g10620.1), *ANS* (Ma05_g03850.1), and bHLH (Ma06_g24180.1) promoters (Figure 7A). Our results indicated that *MaMYB4* represses the anthocyanin biosynthesis in banana, which is likely due to a two-level repression mechanism consisting of (i) reduced expression of anthocyanin synthesis structural genes and (ii) parallel downregulation of the *bHLH* to interfere with the proper assembly of the MYB-bHLH-WD40 activation complex.

## Conclusion

Our data indicate that MaMYB4 acts as a repressor of anthocyanin biosynthesis in banana, which is likely due to a two-level repression mechanism consisting of a reduction of the expression of anthocyanin synthesis structural genes and the parallel downregulation of *bHLH* to interfere with the proper assembly of the MYB-bHLH-WD40 activation complex. To the best of our knowledge, this is the first MYB TF that regulates anthocyanin synthesis that has been identified by the genetic method in bananas, which will be helpful for manipulating anthocyanin coloration in banana plants in the future.

## Data Availability Statement

All sequences generated in this study have been deposited in the National Center for Biotechnology Information Sequence Read Archive (https://www.ncbi.nlm.nih.gov/sra/) with project number PRJNA662185.

## Author Contributions

G-MD and SZ conceived the project. G-MD, SZ, and Q-SY performed most of the experiments. C-YL, G-JY, and F-CB performed the genomic expression analysis. H-JG, TD, and OS performed the RNA-Seq analysis. C-HH and W-DH analyzed the data and wrote the manuscript. All authors read and approved the final manuscript.

## Conflict of Interest

The authors declare that the research was conducted in the absence of any commercial or financial relationships that could be construed as a potential conflict of interest.

## Footnotes

1 https://www.ncbi.nlm.nih.gov/sra/

2 https://banana-genome-hub.southgreen.fr/home1

3 https://www.ebi.ac.uk/Tools/hmmer/

## Supplementary Material

Supplementary Figure 1 The MYBs identified in the banana and clustering analysis of R2R3-MYB in the banana and *Arabidopsis*. **(A)** the number of MYBs identified in banana based on the banana genome and MYB conserved domain (PF00249) from HMMER. **(B)** The conserved domain logo of the R1, R2, and R3 in the banana MYB proteins. **(C)** clustering analysis of R2R3-MYB in the banana and Arabidopsis. A red solid triangle indicates R2R3-MYB was found contain EAR motif and 4 of them also have TLLLRF motif (two red solid triangle). The MaMYB4 (Ma03_g21920.1) is marked red and belongs to subgroup 4.

Supplementary Figure 2 Analysis of MaMYB4 protein domain and expression level of *MaMYB4* in different part of banana leaf. **(A)** MaMYB4 protein contains a conserved R2R3 domain, two EAR motifs (C1 and C2) and a TLLLR motif (C5). **(B)** The purple part (PP) and green part (GP) of banana leaf used for RT-qPCR analysis of *MaMYB4*. **(C)** expression level of *MaMYB4* in different part of banana leaf. Asterisks indicate on the bar indicate significant difference according to Student’s *t*-test (*P*-value < 0.05). Bar indicates standard deviation (*n* = 6).

Supplementary Figure 3 Southern blot analysis of pCMABIA1301:*MaMYB4* transformed banana lines **(A)** and phenotypic characterizations of other three *MaMYB4*-overexpressors **(B)**. DNA fragments containing the 303 bp fragment of the third exon of *MaMYB4* gene were used as a hybridization probe and labeled with digoxigenin. As indicated by the blue arrow, there were 2, 3, 1 and 1 copies T-DNAs in the MaMYB4OE, MaMYB4-2OE, MaMYB4-3OE and MaMYB4-4OE, respectively. M in Lane 1 signify molecular weight marker; P in Lane 2 signify positive control (wild-type DNA); N in Lane 3 signify negative control (empty plasmid pCAMBIA1301 DNA).

Supplementary Data 1 Summary of primers used in this study.

Supplementary Data 2 Metabolome data.

Supplementary Data 3 Transcriptome data.
