# Supplementary figures and images for: MaMYB4, an R2R3-MYB Repressor Transcription Factor, Negatively Regulates the Biosynthesis of Anthocyanin in Banana
Source: Front Plant Sci. 2021 Jan 7;11:600704. doi: 10.3389/fpls.2020.600704 (PMC7817548; doi:10.3389/fpls.2020.600704)

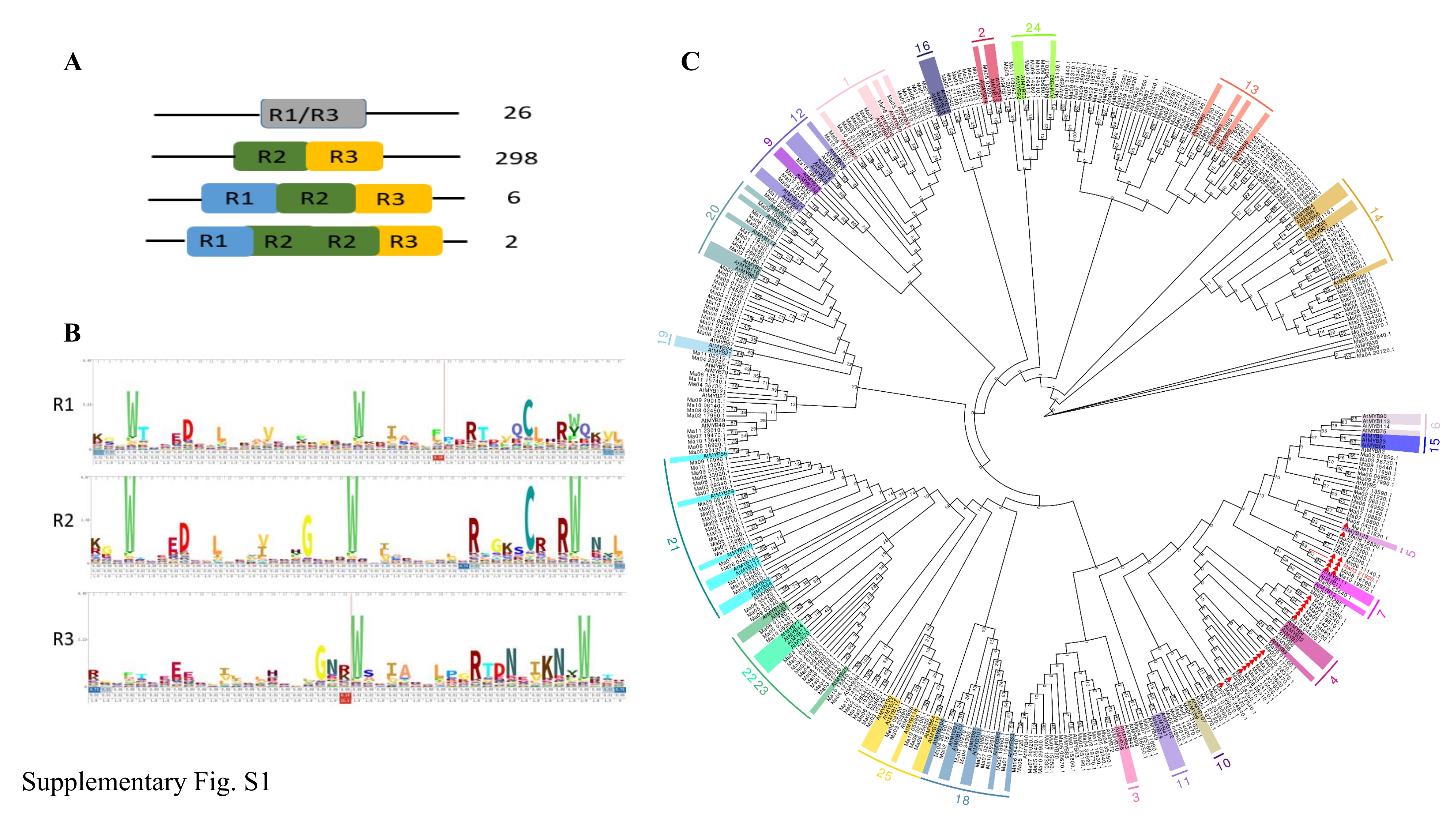

Supplement: Supplementary Figure 1 — The MYBs identified in the banana and clustering analysis of R2R3-MYB in the banana and Arabidopsis. (A) the number of MYBs identified in banana based on the banana genome and MYB conserved domain (PF00249) from HMMER. (B) The conserved domain logo of the R1, R2, and R3 in the banana MYB proteins. (C) clustering analysis of R2R3-MYB in the banana and Arabidopsis. A red solid triangle indicates R2R3-MYB was found contain EAR motif and 4 of them also have TLLLRF motif (two red solid triangle). The MaMYB4 (Ma03_g21920.1) is marked red and belongs to subgroup 4. [file Image_1.JPEG]

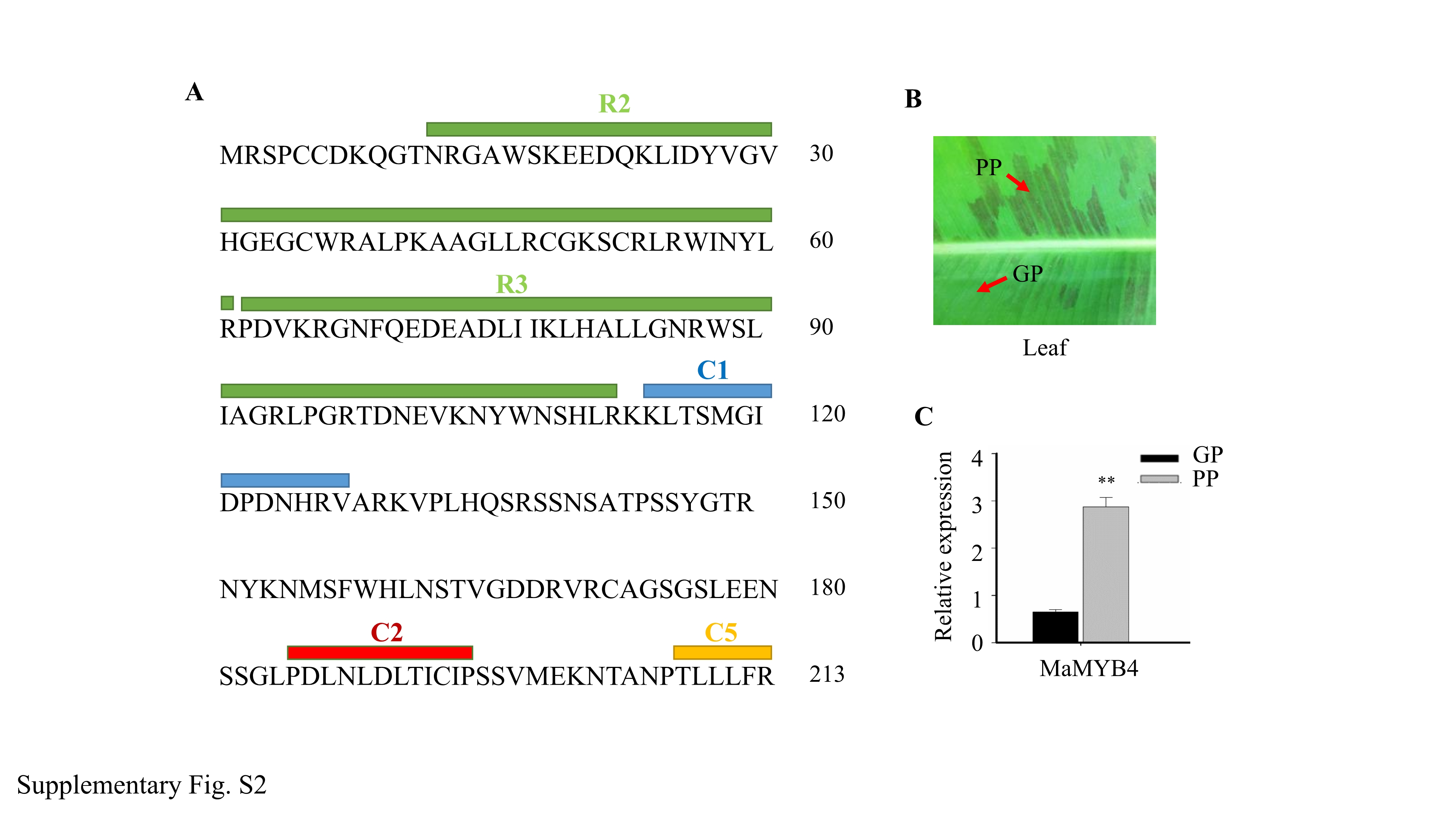

Supplement: Supplementary Figure 2 — Analysis of MaMYB4 protein domain and expression level of MaMYB4 in different part of banana leaf. (A) MaMYB4 protein contains a conserved R2R3 domain, two EAR motifs (C1 and C2) and a TLLLR motif (C5). (B) The purple part (PP) and green part (GP) of banana leaf used for RT-qPCR analysis of MaMYB4. (C) expression level of MaMYB4 in different part of banana leaf. Asterisks indicate on the bar indicate significant difference according to Student’s t-test (P-value < 0.05). Bar indicates standard deviation (n = 6). [file Image_2.JPEG]

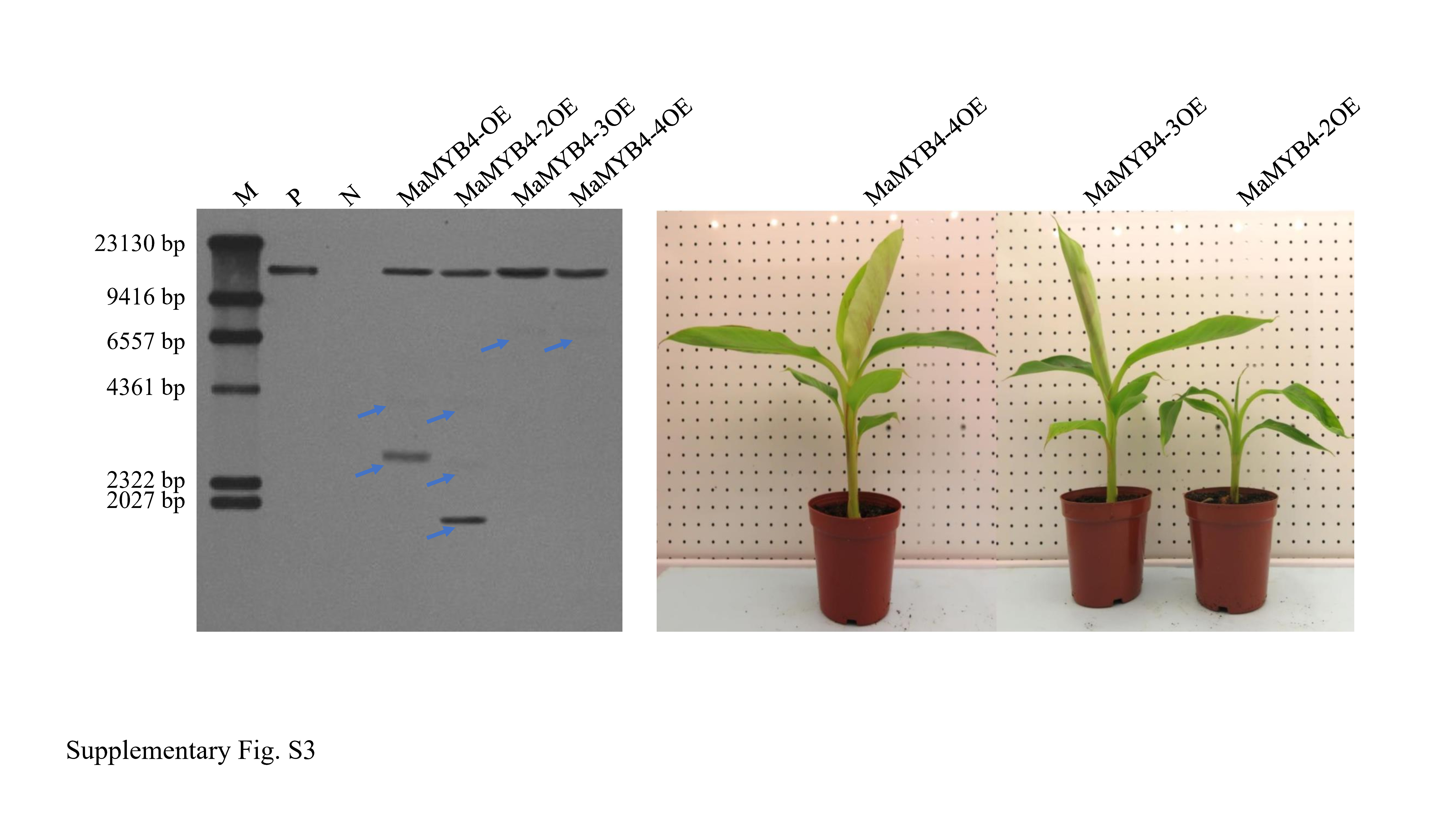

Supplement: Supplementary Figure 3 — Southern blot analysis of pCMABIA1301:MaMYB4 transformed banana lines (A) and phenotypic characterizations of other three MaMYB4-overexpressors (B). DNA fragments containing the 303 bp fragment of the third exon of MaMYB4 gene were used as a hybridization probe and labeled with digoxigenin. As indicated by the blue arrow, there were 2, 3, 1 and 1 copies T-DNAs in the MaMYB4OE, MaMYB4-2OE, MaMYB4-3OE and MaMYB4-4OE, respectively. M in Lane 1 signify molecular weight marker; P in Lane 2 signify positive control (wild-type DNA); N in Lane 3 signify negative control (empty plasmid pCAMBIA1301 DNA). [file Image_3.JPEG]
